# A qualitative analysis of participant experiences with universal school-based depression screening

**DOI:** 10.1016/j.pmedr.2022.102073

**Published:** 2022-11-29

**Authors:** Deepa L. Sekhar, Elizabeth Hivner, Alissa Molinari, Kathleen Allen, Heather Stuckey

**Affiliations:** a Department of Pediatrics, Penn State College of Medicine, 500 University Drive, HS83, Hershey, PA 17033, United States; b Department of Medicine, Penn State College of Medicine, Hershey, PA, United States

**Keywords:** Depression screening, Adolescent health, School health

## Abstract

Screening in High Schools to Identify, Evaluate and Lower Depression (SHIELD) was a randomized clinical trial of school-based universal depression screening conducted with public high schools in Pennsylvania, United States. Screened adolescents were twice as likely to initiate depression treatment compared to peers who were not screened. The purpose of this qualitative study was to understand the experience of communities participating in universal screening via SHIELD. Semi-structured interviews were conducted with school staff (n = 11), parents (n = 4) and adolescents (n = 7) from April-October 2021. A codebook was developed with 20 % of audio transcripts coded for inter-rater reliability (staff k = 0.88, parents k = 0.90, adolescent k = 0.74). The two coders divided and individually coded remaining transcripts for thematic content analysis. Three themes were identified: 1) Depression screening was well-received; 2) The main challenge with screening was inadequate staffing to manage referrals; and 3) School staff suggested alternate formats and methods for future screening. In summary, school-based depression screening was well-received, but schools experienced challenges with staffing and resources to support identified adolescents and offered alternative suggestions for future screening. Schools interested in depression screening need education and capacity building focused on barriers and challenges with the process. Learning from staff who have realized this evidence-based practice in their respective districts will support next steps towards implementation.

## Highlights

• School-based depression screening was well-received by participating communities.
• The main barrier was adequate staffing to manage screening referrals.
• Schools planning implementation need support focused on anticipated challenges.

## 1 Introduction

The prevalence of adolescent major depressive disorder (MDD) has increased by greater than 70 % from 8.3 % in 2008 up to 14.8 % in 2018 (Healthy People.gov, 2021). Paralleling this rise, suicide is currently the second leading cause of death among United States (US) adolescents (Centers for Disease Control and Prevention. National Center for Health Statistics. Leading Causes of Death, 2022). These statistics were further worsened by the recent COVID-19 pandemic (Racine et al., 2021, Jones et al., 2022). While the US Preventive Services Task Force endorses adolescent MDD screening in primary care, the majority of US adolescents do not present for routine preventive care (Siu, 2016, Irwin et al., 2009, Rand and Goldstein, 2018). Recognizing that schools already conduct physical health screenings (e.g., vision, hearing) to identify barriers to student academic success, the “Screening in High Schools to Identify, Evaluate and Lower Depression” (SHIELD) study evaluated the effectiveness of adolescent school-based screening for MDD (Sekhar et al., 2021, Sekhar et al., 2019).

SHIELD was a randomized clinical trial (RCT) conducted from November 2018–2020 (Sekhar et al., 2021, Sekhar et al., 2019). SHIELD compared the usual school practice of targeted screening based on observable behaviors of concern to universal screening for adolescent MDD using the Patient Health Questionnaire-9 (PHQ-9) (Sekhar et al., 2021, Sekhar et al., 2019, Richardson et al., 2010). Fourteen Pennsylvania public high schools and 12,909 adolescents participated (median age 16 years [range, 13–21]; 6,963 [53.9 %] male), of whom 7,067 (54.7 %) were Hispanic, non-Hispanic Black or multiracial/other (Sekhar et al., 2021). A total of 6,473 (50.1 %) adolescents were randomized to universal screening, and these adolescents had 5.9 times higher odds (95 % CI 5.1–6.9) of being identified with MDD symptoms and 2.1 times higher odds (95 % CI 1.4–3.1) of initiating MDD treatment (Sekhar et al., 2021).

There is precedent for schools to conduct universal mental health screening (McCormick et al., 2009). An RCT by Husky, et al. of four Pennsylvania high schools demonstrated increased treatment initiation with screening (Husky et al., 2011). In contrast, Guo, et al.’s RCT in California middle schools found no effect of universal screening on treatment initiation (Guo et al., 2017). This may be due to a smaller sample size, younger students, a large percentage of Asian students (historically less likely to engage in mental health treatment), and different positive screen criteria (Guo et al., 2017, Lipari et al., 2013, Alegría et al., 2008, Yang et al., 2020).

Understanding that adolescent depression and mental health can be a complicated and stigmatizing topic (Clement et al., 2015), the success of SHIELD depended greatly upon engagement with key stakeholders and participating schools (Hoke et al., 2022). Prior to screening for the RCT, the SHIELD team conducted focus groups with parents and adolescents at participating schools to understand knowledge and attitudes regarding mental health, depression and the acceptability of school-based screening (Stuckey et al., 2021). Parents and adolescents concurred that depression was a serious issue and that schools should be involved, but raised concerns regarding consent and communication of results. Few focus group participants were aware of existing school resources to support mental health or what steps to take if an adolescent were identified with depression (Stuckey et al., 2021).

After the study, the team went back to participating communities to better understand the experience of the screening. Prior work supports that school-based depression screening is acceptable to parents and adolescents and that schools are willing to partner in this space (McCormick et al., 2009, Husky et al., 2011, Guo et al., 2017, Stuckey et al., 2021, Sekhar et al., 2021). However, the SHIELD study provided a unique opportunity to evaluate the experience of school-based depression screening with those who directly participated in the process.

The main study purpose was to understand the barriers and successes related to the screening process from the perspectives of school staff, parents and adolescents who participated in universal screening. Specifically, the research team sought to understand what individuals recalled from the experience, what elements were handled well, what pieces of the screening process needed improvement, and if participating schools planned to continue universal depression screening independently of the SHIELD study.

## 2 Materials and methods

### 2.1 Study design

Semi-structured interviews were conducted with all participants. This format was selected to protect confidentiality, as some participants may have had a positive PHQ-9 screen requiring treatment and be reluctant to discuss this in a focus group. Interview guide questions were developed and refined by the research team. The parent interview guide questions were updated after completing three interviews, and the adolescent interview guide after facilitating five interviews due to recognition by the research team that most individuals had little recollection of completing the PHQ-9, especially if the screen was negative. With a negative PHQ-9 screen, any follow-up questions regarding the referral process could not be answered by the interviewees. The interview guides were revised to allow for more hypothetical questions, e.g., “If the screen were positive, what would you expect should happen?” Both versions of the parent and adolescent interview guides are included as appendices (A and B). However, only the second or “B” version of the parent and adolescent transcripts were used in the current analysis. This study was approved by the Penn State College of Medicine Institutional Review Board.

### 2.2 Recruitment

Participating SHIELD study high schools sent an electronic message and flyer to eligible high school adolescents and parents, similar to previous strategies (Stuckey et al., 2021). Eligibility criteria required that participants be an adolescent, or parent/guardian of an adolescent, enrolled in a participating high school and in a grade randomized to the universal screening arm of the SHIELD RCT.

To determine eligibility, parents completed a brief screener in Research Electronic Data Capture (REDCap), a secure, web-based application for research study support (Harris et al., 2009 Apr). Parents completed the demographic questionnaire for students under 18. Completion indicated the parent’s consent for student participation and student’s implied consent/assent. Students over 18 completed the demographic questionnaire themselves.

For students under 18, a study coordinator scheduled the interview by emailing the student and copying the parent. For students over 18, the study coordinator emailed only the student. All interviews occurred virtually via zoom. Interviews were conducted individually, even for students under 18. Parents were not required to be home during the interview if their student was underage. Adolescents (<18 years old) provided assent before starting the interview in addition to parental consent documentation. The study team did not develop a protocol for medical or psychological resources; risks involved in participating were low and questions were not sensitive.

An electronic message was also sent to school staff for participation. Eligibility criteria required that school staff had been involved with the SHIELD study and universal MDD screening at their respective schools. Upon screening eligible, and providing consent and contact information, a study team member reached out to request completion of an online demographic questionnaire and to schedule the interview.

### 2.3 Interview process

The 30-minute zoom-audio interviews were conducted from April-October 2021 by AM and KA after receiving training from a qualitative expert (HS). After brief introductions, background information, and time for questions, the participant was instructed to turn off their camera so that only the audio recording was captured. The interviewee notified the participant when the recording began and followed the semi-structured facilitator guide. The recorded audio files were transcribed by a professional service, from which the project team members reviewed the transcripts and imported them into a qualitative software management system (Nvivo 12 Plus).

### 2.4 Analysis

The study used qualitative description as described by Sandelowski, et al. (Sandelowski, 2000, Sandelowski, 2010). A purposive sampling technique was used to gain the perspectives of those who experienced the intervention. Codes were systematically applied in an inductive manner, generated from the data themselves in the course of the interpretation of the data (Sandelowski, 2000). The codebook was created by reading through all of the data, collecting a summary of the investigators’ initial individual codes and then synthesizing all codes into a draft codebook, which was agreed upon by all investigators. 

A portion of the transcripts (20%) of each group were coded for inter-rater reliability (staff k = 0.88, parents k = 0.90, and adolescents k = 0.74), and then two coders divided and completed coding of the remaining transcripts individually. Team members determined themes by reviewing the codebook and grouping categories into larger overarching themes.

## 3 Results

Participants came from 4 of the 14 schools involved in the SHIELD RCT. As shown in Table 1, school staff (n = 11), parent (n = 4), and adolescent (n = 7) participants were (respectively) white (82%, 75%, 43%) and female (91%, 75%, 100%). The majority of school staff were educated at a graduate degree level (91 %), and the majority of parents at a high school level (50 %). The study team identified three primary themes from the codebook that related to the barriers and successes of the school-based depression screening (Table S1).

**Table 1:** Demographics of Adolescent, Parent, and School Staff Interview Participants.

Characteristics	School Staff Participants (n = 11)		Parent Participants (n = 4)		Adolescent Participants (n = 7)		
	n	%	n	%		n	%
**Sex**							
Female	10	**90.9**	3	**75.0**		7	**100.0**
Male	1	**9.1**	1	**25.0**		0	**0.0**
Other	0	**0.0**	0	**0.0**		0	**0.0**
**Mean age**					**Age**		
30–39	3	**27.27**	0	**0.0**	14	0	**0.0**
40–49	2	**18.18**	2	**50.0**	15	1	**14.3**
50–59	4	**36.36**	1	**25.0**	16	5	**71.4**
60+	2	**18.18**	1	**25.0**	17	1	**14.3**
					18	0	**0.0**
**Race**							
White	9	**81.81**	3	**75.0**		3	**42.9**
Black or African American	1	**9.09**	0	**0.0**		1	**14.2**
Asian	1	**9.09**	**1**	**25.0**		3	**42.9**
Native Hawaiian or other Pacific Islander	0	**0.0**	0	**0.0**		0	**0.0**
American Indian or Alaska Native	0	**0.0**				0	**0.0**
More than one race/ ethnicity	0	**0.0**	0	**0.0**		0	**0.0**
**Hispanic**							
No	11	**100.0**	4	**100.0**		7	**100.0**
Yes	0	**0.0**	0	**0.0**		0	**0.0**
**Education**					**Grade**		
High school graduate (or equivalent)	0	**0.0**	2	**50.0**	9	1	**14.3**
Associate’s Degree	0	**0.0**	0	**0.0**	10	4	**57.1**
Bachelors’ Degree	1	**9.10**	1	**25.0**	11	2	**28.6**
Master’s Degree	10	**90.90**	1	**25.0**	12	0	**0.0**

Theme 1: The PHQ-9 depression screen was well-received by school staff, adolescents and parents.

School staff shared positive comments about the school-based depression screening process, and that mental health “plays a huge role in daily life,” and the screening sets them up “for a good situation from now into their…future.” Staff commented that parents were “quite pleased” that staff were able to identify that their child had a need.

*…If students learn now at this age level, how to handle anxiety, stress, depression at this level, it can only benefit them in the future*

*…Mental health has…for far too long, been shoved under the carpet or hidden in the closet. People don't want to talk about it and it needs to be talked about…*

Parents believed the school was a good environment to screen, and school should be involved in the follow-up of identified adolescents. It was important that their student have someone with whom they could confide if there was a problem, and school was a place to allow adolescents to raise mental health issues with adults other than their parents. Parents would prefer notification of positive screen results by phone and would appreciate the school providing some resources to assist them in getting their adolescent help, whether a guidance counselor or a social service agency. Several interviewees had already gone through this process with the school for their other children and reported overall positive experiences.

*I know my kids, sometimes they feel better talking to somebody else than me. And that's fine as long as they get help.*

Parents indicated they would talk to their adolescent about the results, as a first step to “understand what’s going on.” They would also reach out to their pediatrician or family physician in their network for assistance in securing help for their adolescent.

Adolescents were comfortable with the school counselor having the screening results. They identified others, e.g. siblings and teachers, whom they would preferentially involve prior to parents, though most ultimately were accepting of parental notification. As a form of follow-up treatment, students were more comfortable with receiving counseling than medication.

*Not that we have anything against medication, but I just feel like trying to understand what you're going through first is important before this prescription.*

*I feel like a lot of adults might freak out when they hear, “Oh, your child might be dealing with something like that.” And they might automatically go to medication because they want the problem solved right away, but that's not always the best thing to just rush into stuff like that because medication isn't a small thing.*

Adolescents were more interested in counseling outside of school or after school hours to avoid being seen or having others know that they see a counselor.

Theme 2: The main challenge with screening was inadequate staffing to manage referrals.

While logistics on the screening day were efficient and clear, school staff shared the challenge of inadequate staffing..

*The biggest challenge on the day of the screening was…getting the results back and then making sure we had enough people to follow up with the students that were… [an]…immediate threat to themselves. There was a long list of them. And there was only so many of us that were available while also managing the rest of the guidance suite.*

Schools planned their own processes for following up with students identified at risk based on the screening. One staff member recalled that two adolescents were hospitalized after the screening. Several schools decided to have a maximum number of positives for suicide risk based on PHQ-9 item number nine, after which screening stopped for the day.

*When we hit 10 to 12, that's when we called the screener off for the day, because we were…overwhelming our mental health provider that was assisting us. Even though we had three or four of them at a time, it was overwhelming because by the time they did the whole process, each screener covered two to three kids throughout the day, which…would take…sometimes several hours.*

Additional challenges were adolescent question comprehension, e.g., some did not notice the screener referred to how adolescents felt in the past two weeks. School staff mentioned that after adolescents understood their positive responses would lead to being called to the office for further discussion, some may have answered dishonestly to avoid this. Staff also discussed the difficulty in contacting parents and getting individuals to follow through with treatment recommendations. However, many of the identified adolescents were already in treatment and/or known to the school.

*Typically,…our school has…an overall struggle…getting people to follow through with the services, but many of those kids that showed up on the screening are either already receiving services, or it was like a misunderstanding of…not misunderstanding, but just the way it's worded, they answered it, “yes”, and it wasn't really exactly what they were thinking.*

*…I did know that a lot of kids came out of the woodwork, so to speak, with elevated levels of anxiety and obviously depression.*

Theme 3: School staff suggested alternate formats and methods for future screening.

School staff recognized a need for ongoing screening. In trying to navigate the pandemic, staff saw “a lot of issues with mental health, isolation concerns, anxiety.” Adolescents had to deal with loss of life due to COVID and an extraordinary amount of grief.

*…Our ninth graders, for example, they spent their eighth grade year at home. Their level of maturity may not be where it needs to be. They came from a home environment. Now they're in a very demanding high school, depending on their academic program. Some people handle things better than others, but I would say the state of the world is affecting us locally and directly in the schools, and that's why we still need…the depression screener.*

Despite the need, staff were unsure about specific plans to continue the screening in future years. In some cases, staff shared general future screening plans that would be broader than depression alone (e.g., including anxiety and substance use). It was suggested screening might also be altered to link with a broader social-emotional curriculum for adolescents.

*…We're not using the survey that you used … but…[use]…data so we can call in a speaker or do some sort of school-wide tier one intervention training with teachers.*

*…We're going to be basing it [the screener] on that [depression] as well as if there's some sort of drug and alcohol use, or if you're having difficulty in your classes, how do you deal with if you're having an argument with your friend, that kind of stuff. Not specifically directly for depression.*

Staff shared that administrative turnover meant those currently in charge were unaware of prior efforts in this space. Thus, even while they had an interest in continuing, the information had to be vetted with the new administration.

*…With the change of leadership…there were other things they were trying to get situated, but I don't think it's a bad idea to implement that. Just to be aware, it almost red flags the kids, to keep an eye on certain kids.*

## 4 Discussion

A series of follow-up interviews with school staff, parents and adolescents who participated in the SHIELD RCT universal screening arm suggested that school-based depression screening was overall well-received. Confidentiality, raised in the prior focus groups (Stuckey et al., 2021), was a concern that was reiterated by adolescents who participated in the screening. Finally, the value of school involvement in mental health was recognized, especially in the context of the COVID-19 pandemic, but school staff raised concerns about staffing and resources and did not articulate clear plans for how they would continue screening moving forward.

Parents and adolescents had little recollection of the universal school-based depression consent and screening process itself, which may be related to recall bias. SHIELD used an opt-out consent process (Sekhar et al., 2021, Sekhar et al., 2019). The concept of recall bias traditionally has been applied to case-control studies in which cases, e.g. cancer patients, are much more likely to recall a toxic exposure than healthy controls (Coughlin, 1990, Tenny et al., 2021.). The concept translates similarly to the current study, as those who did not have a significant outcome, e.g. a positive depression screen, had limited recollection of the screening opt-out forms and administration of the PHQ-9. Considering the stigma often attached to mental health (Clement et al., 2015), it is reassuring that none of the individuals interviewed expressed anger or indignation over the consent and screening process.

Similar to the previous focus groups conducted for the SHIELD study (Stuckey et al., 2021), adolescents expressed concerns about confidentiality. However, adolescents were comfortable with the school staff receiving their screening results, which supports the role of the school in the management of adolescent mental health. Adolescents were more concerned about disclosure of positive results to parents, and were clear that they (the adolescents) should be notified first and have the opportunity to discuss the situation with school staff. A prior national survey of parent opinions regarding school-based depression screening indicated that 93% wish to be informed of a positive result (Sekhar et al., 2021). The balance between providing adolescents a safe space to disclose and informing parents remains an ongoing challenge, as some adolescents who would benefit from help may hesitate to disclose symptoms based on the concern their parents will be approached (Hoover and Bostic, 2021). School staff similarly raised the issue that adolescents might not answer the screen honestly due to concerns about the follow-up and parental involvement.

Despite the positive aspects of school-based depression screening, screening was a significant task for participating schools. Different from physical health screens, a treatment delay for depression may have more significant consequences. School staff agreed on the value of screening, but how to implement and sustain depression screening was not clear. Compared to the healthcare sector, even well-resourced schools struggle from a significant implementation gap with regard to evidence-based practices (Bhatta et al., 2018, Cook et al., 2019). At the conclusion of the RCT, it was recognized that schools who participated in the SHIELD study were those who felt adequately equipped to manage an increased number of referrals with screening (Sekhar et al., 2021). Yet, even among these schools who successfully participated in the SHIELD universal screening, additional supports are needed for sustained, successful implementation of universal depression screening.

Specifically, schools need clear screening guidelines (e.g., consent procedures, frequency). Resource mapping can be used to identify additional school and community-based supports. Also, professional development can expand the capacity of existing staff to manage screen positive students. All identified students do not require psychiatric services, and for some a one-to-one check in with a school counselor or parental notification regarding positive symptoms with recommendation for primary care follow-up will be sufficient. Additional implementation supports for screening logistics, e.g., the number of students per day, where and when in the building to screen, and addressing stigma from a positive screen also require thoughtful discussion and planning.

## 5 Strengths and limitations

The results reported here have several limitations. Those who chose to participate in the interviews may have held more favorable views on the topic of depression and school-based screening. Acknowledging the well-documented disparities in mental health for minority populations (Stewart et al., 2012, Georgiades et al., 2018, Merikangas et al., 2011), inclusion of interviews from underrepresented populations would be insightful. Similarly, most participants identified as female. Those who identify as male or non-binary may have had a different experience with screening that was not captured here.

Like many other studies conducted during this time, the COVID-19 pandemic and its impact on school closures and the mental health of adolescents likely influenced our findings (Singh et al., 2020). Depression screenings ended in March 2020 due to the pandemic (Commonwealth of Pennsylvania. Governor Wolf Announces Closure of Pennsylvania Schools, 2020). Five student and three parent interviews were conducted in July 2020 after which limited recollection of the screening process prompted revision of the interview guide. Due to school challenges with reopening in fall 2020; the remaining interviews were not conducted until April to November 2021; this time lag may have further impacted recall.

Finally, though the participants were a unique sampling of school staff, parents and adolescents who participated in the MDD screening, the overall sample size was smaller than planned and included four of the 14 SHIELD schools. Saturation is difficult to determine because it is the prediction of the unobserved based on the observed (Saunders et al., 2018); however, based on the data we collected from participants, we were able to capture a reasonable range of experiences to address the research question (Thorne, 2008).

## 6 Conclusion

The results of this qualitative analysis indicate that school-based depression screening is viewed favorably by communities who have participated in this process. Yet, the qualitative data indicate that additional education and capacity building will be required for schools to independently sustain implementation of this evidenced-based practice. Beyond existing toolkits of available resources (National Center for School Mental Health [NCSMH], 2020) additional steps to facilitate implementation may include bringing together school staff with peer schools who have successfully realized school-based depression screening. This “train the trainer” model has been successfully implemented in other evidence-based practices with communities (Triplett et al., 2020). Involving parents and adolescents in the discussion and process development may mutually support community-education and stigma reduction, and successful implementation. This qualitative study is unique in obtaining feedback on universal depression screening from those who were direct participants in the process and setting the stage for next steps towards implementation.

## CRediT authorship contribution statement

**Deepa L. Sekhar:** Conceptualization, Methodology, Formal analysis, Writing – original draft, Writing – review & editing, Visualization, Supervision, Project administration, Funding acquisition. **Elizabeth Hivner:** Methodology, Software, Formal analysis, Investigation, Data curation, Writing – original draft, Writing – review & editing, Visualization, Project administration. **Alissa Molinari:** Software, Formal analysis, Investigation, Data curation, Writing – original draft, Writing – review & editing, Visualization. **Kathleen Allen:** Software, Formal analysis, Investigation, Data curation, Writing – original draft, Writing – review & editing. **Heather Stuckey:** Methodology, Software, Validation, Formal analysis, Resources, Data curation, Writing – review & editing, Supervision, Project administration.

## Declaration of Competing Interest

The authors declare that they have no known competing financial interests or personal relationships that could have appeared to influence the work reported in this paper.

## Data Availability

Data will be made available on request.
