# Supplementary material for: A qualitative analysis of participant experiences with universal school-based depression screening
Source: Prev Med Rep. 2022 Nov 29;31:102073. doi: 10.1016/j.pmedr.2022.102073 (PMC9722442; doi:10.1016/j.pmedr.2022.102073)
Supplement: Supplementary data 1 [file mmc1.docx]

**Interview Guides**

School Staff

Thank you for agreeing to participate in this interview. During the past school year, your high school partnered with our team and the Patient Centered Outcomes Research Institute (PCORI) to conduct a depression screener for select grades. We believe that getting your opinion will make the study better.

The goal of this discussion is to hear your thoughts and opinions about the topic of the depression screening process at your school. We are also going to ask questions about your thoughts about continuing the screening process without our team. You may decline to answer any questions that you may not want to answer. Anything said here will remain confidential. If you do share a story that we feel puts you or a student in immediate danger or if we have concern for the safety of either of you, we are required to report that to someone who can help you. Do you have any questions? If not, I will begin the recording.

*Begin audio recording*

1. Does your school have plans to independently continue the depression screener?
   1. If no – why not?
2. How was the tracking/reporting students?
3. Many staff indicated to us in the past that our team’s support and communication (i.e. technical assistance) was effective at helping your school facilitate the screener. Describe which components of the assistance was the most helpful.
   1. Webinar at the beginning of the year
   2. Regular support
   3. Help day-of only
   4. Follow-up tracking support
4. If we could adjust the screening tool or process, what would help?
   1. How did you handle the students who were flagged as suicidal?
5. If your school was to do the screener independently next year, what would you need to change or adopt to be successful?
   1. What methods for communication internally to teachers/admin were successful?
   2. What methods were not successful?
6. What parts of the screening process did not go as expected? How might you plan for that in the future?
7. What parts of the screening process would be challenging to do without our team? Why?
8. How successful do you feel the school student assistance program is in identifying students in need and successfully connecting them to services?
   1. What could be done differently?
9. For the students who need additional support internally from the school, what types of resources/support would your school need in order to serve these students more effectively?
10. Would you like to share any final thoughts on anything we discussed today?

*Stop audio recording*

Thank you so much for your time. In the event that you have any follow up thoughts, you can reach out to me by email to share your thoughts. I will provide you with my email so you can contact me.

Parent Version A

Thank you for agreeing to participate in this interview. During the past school year, your child’s high school partnered with our team and the Patient Centered Outcomes Research Institute (PCORI) to conduct a mood screener for select grades. We believe that getting your opinion will make the study better.

The goal of this discussion is to hear your thoughts and opinions about the topic of the mood screener process at your school. Anything said here will remain confidential. If you do share a story that we feel puts you or your child in immediate danger or if we have concern for the safety of either of you, we are required to report that to someone who can help you. Do you have any questions? If not, I will begin the recording.

*Begin audio recording*

1. During the 2019 – 2020 school year, your student was in one of the grades that completed a mood screener. Do you recall receiving a letter about this process?
   1. If yes, was this method of communication, which allowed you to opt-out your child helpful and clear?
2. Was the mood screener discussed or mentioned by other parents? If so, how was this talked about?
3. Did your student mention the mood screening to you?
4. Would you want your child screened each year?
5. Are you aware of the student assistance program or SAP team? If so, what is your impression of them?
6. Is there anything else you would like to add before we end this interview?

Parent Version B

Thank you for agreeing to participate in this interview. During the past school year, your child’s high school partnered with our team and the Patient Centered Outcomes Research Institute (PCORI) to conduct a depression screener for select grades. We believe that getting your opinion will make the study better.

The goal of this discussion is to hear your thoughts and opinions about the topic of the depression screener process at your school. We are also going to ask scenario questions about what you would do if your child were depressed or experiencing negative mental health symptoms. You may decline to answer any questions that you may not want to answer. Anything said here will remain confidential. If you do share a story that we feel puts you or your child in immediate danger or if we have concern for the safety of either of you, we are required to report that to someone who can help you. Do you have any questions? If not, I will begin the recording.

*Begin audio recording*

1. For this first question, I don’t need any particular details, just looking for a yes or a no and this would just be for my frame of reference. Do you have any personal experience with a family member, friend or yourself dealing with a mental illness?
2. During the 2019 – 2020 school year, your student was in one of the grades that completed a depression screener. Do you recall hearing anything about the screener from the school, your child or other parents ?
   1. If parent doesn’t remember, provide a description of depression screener - the type of questions, and the process (e.g., our team got approval from your school to complete this process in the selected grades, went in with iPads, confidential from study team but not the school, etc.).
3. Do you think that a school setting is an appropriate place to ask questions about depression on a school-wide scale?
4. So, let’s say that the depression screening that your child completed indicated that they might be depressed, what do you think the school should do next? Or if this type of situation has happened to you, would you be willing to share your experience? Were you happy with how the situation was handled or is there something you wish the school would have done differently?
   1. Would you want to be notified? How would you want to be notified (email, call, text)? When?
5. So, after you have been notified from the school that your child might be depressed, what would be your first step be?
   1. What do you think should happen?
   2. Prompting for additional steps if they give little info
6. If the depression screening indicated that your child might be suicidal, what do you think the next step is for the school to do? Or if you actually were notified by the school last school year, with a concern about a child who may be suicidal, was there anything that you wish the school would have done differently?
   1. Would you want to be notified? How would you want to be notified (email, call, text)? When?
7. After hearing from the school that your child may be suicidal, what would your first step be?
   1. What do you think should happen?
   2. Prompting for additional steps if they give little info
8. Separate from the depression screener, imagine you yourself noticed that your teen was acting depressed, not going out with friends as much, more moody than usual, sleeping a lot more, and you were concerned about depression. Would you try to have a conversation about this, and what would that look like? (If you would not have a conversation, what would you do?)
   1. How would you open up that conversation? What words would you use?
   2. When might that conversation take place?
   3. Where might that conversation take place?
   4. Would you talk to them alone? Or with another parent/adult/physician?
   5. What if they denied and said they were fine? What happens then?
9. So let’s say that your child admits to you that they have been having a hard time and don’t know what to do. What are your thoughts on treatment for depression? What would your first step be?
   1. When people think about treating asthma, they might picture an inhaler.
   2. When you think about “treatment” for depression, what do you picture?
   3. What treatments would be comfortable to you and your child?
   4. How long do you anticipate it takes to get better?
10. One of my goals is to understand what parents might be saying and thinking when approaching their child about depression so that we are able to give tools and resources may be helpful for parents.
    1. With this in mind, are there any particular resources or tools that you think would be helpful for you and other parents/guardians to have when approaching your child for this type of conversation?
11. Is there anything else you would like to add before we end this interview?

Student Version A

Thank you for agreeing to participate in this interview. During the past school year, your high school partnered with our team and the Patient Centered Outcomes Research Institute (PCORI) to conduct a mood screener for select grades. We believe that getting your opinion will make the study better.

The goal of this discussion is to hear your thoughts and opinions around the topic of the mood screener process at your school. Anything said here will remain confidential. If you do share a story that we feel puts you in immediate danger or we become concerned for your safety, we are required to report that to someone who can help you. Do you have any questions?

*Begin audio recording*

1. During the 2019 – 2020 school year, your class took a mood screener. Can you tell me if you felt it was taken seriously or as a joke by your peers?
2. What was your reaction to the screener?
   1. Were the instructions clear?
   2. Did you understand that the questions were specifically asking how you felt in the previous 2 weeks?
   3. Did you feel like you could be honest in your responses?

Do you think these questions are okay to ask at school, or would you feel better about completing it at a doctor visit? Why or why not? Even if your parent were present?

1. If the mood screening that you completed indicated that you might be depressed, what did you think would happen next?
2. What do you think should happen?
3. Students with a concerning mood screen were referred to the student assistance program to determine if they were in need of additional help. In general, how do students at your school view the student assistance program?
4. Were the mood screener instructions clear?
   1. Did you understand that the questions were specifically asking how you felt in the previous 2 weeks?
5. Are there specific concerns regarding mood/mental health you have for yourself or your peers when you think about returning to school following the coronavirus pandemic?

Student Version B

Thank you for agreeing to participate in this interview. You may be aware that your high school worked with our team to administer a depression screening for some students at your school during the 2019/2020 school year. Students were asked to complete the screener on iPads in your school building. My hope for our conversation is to hear your thoughts on the process that your school uses to follow-up with the students who may need a referral for mental health due to the screening. You may be familiar with this group, as they are generally called the student assistance program and usually include a team of counselors and other staff.

A few things before we get started:

1. I will be recording our conversation so that I can access what you shared with me at a later time. Your name and the specifics of what you share will remain confidential, but my team will be taking a look at all of the information shared with us during multiple interviews and developing a summary to share with others.
2. If you do share a story that I feel puts you in immediate danger or I am concerned for your safety, I am required to report that to someone who can help you.
3. You may decline to answer any questions that you may not want to answer.
4. Do you have any questions?

*Begin audio recording*

1. As I mentioned before, our team was at your school in the 2019/2020 school year to facilitate a depression screening for students. These are the questions that were asked *[Share screen and show screening questions]*
   1. Does this screening tool look familiar?
   2. (If yes) How comfortable did you feel completing this last year?
   3. (If no) Describe – these are the questions we used last year at your school and they relate to feelings of depression.

*For the rest of our conversation when I refer to the depression screener, this is what I’m referring to*.

1. I don’t need any particular details, just looking for a yes or a no and this would just be for my frame of reference. Do you have any personal experience with a family member, friend or yourself dealing with a mental illness?
2. Let’s consider a hypothetical situation: If *you* answered these screening questions in school and your results showed that you may be depressed, what would you like to happen next? (I’m not asking what should happen or what currently happens, but what your preference would be)
   1. Who would you trust to see the screening results?
      1. Prompt: parents, counselors, teachers?
   2. What would you want this person to do with the results?
   3. Should the school contact anyone else?
      1. If yes, who would you want them to contact?
3. Keeping with this same scenario, who would you feel most comfortable chatting with about your screening?
   1. Prompt: parents, counselors, teachers, friends?
4. What topics would you want to be talked about in this conversation with your trusted person?

Note – allow participant to answer open-endedly first, then offer list under b.

- 1. Taking into account timing, environment, word choice, formal/casual, etc.
  2. Would you want to talk about:
     1. Counseling
     2. Medication
     3. Social supports (family, friends, trusted teachers, etc.)
     4. Resources (phone numbers, apps, tools that they could use, etc.)
     5. Recent stressful events
     6. Symptoms of depression
     7. Activities/interactions with friends

1. Separate from the depression screener, imagine you were feeling depressed, not going out with friends as much, more moody than usual, sleeping a lot more and your parent (or other adult that you live with) was concerned about you. Do you think your parent (or another adult that you live with) would try to have a conversation about it with you, and what would that look like? If your parent (or another adult that you live with) would not have a conversation with you, what would you do?)
   1. How would you like your parent (or other adult that you live with) to open up that conversation? What words would you like them to use?
   2. When would you like that conversation take place?
   3. Where would you like that conversation to take place?
   4. Would you like them to talk to you alone? Or with another parent/adult/physician?
   5. What if you denied and said you were fine? What happens then?
2. So let’s say that you admit to your parent (or other adult that you live with) that you have been having a hard time and don’t know what to do. What are your thoughts on how that individual would take steps towards treatment for your depression? What would their first step be?
   1. When people think about treating asthma, they might picture an inhaler.
   2. When you think about “treatment” for depression, what do you picture?
   3. What treatments would be comfortable to you and your parent (or other adult)?
   4. How long do you anticipate it takes to get better?
3. Okay now that we know what you might want follow-up and conversation to look like, let’s go back to what your school’s process currently is. Most likely your school would meet with you first and then contact your parents to determine if school counseling or outside counseling was the best option. How does knowing that make you feel?
   1. If this happened to you, how would you react?
   2. How would you feel about your parents knowing this information?
   3. Would you prefer going to counseling at school or outside of school? Why?
4. Do you have any additional comments or questions?
