# Supplementary material for: A qualitative analysis of participant experiences with universal school-based depression screening
Source: Prev Med Rep. 2022 Nov 29;31:102073. doi: 10.1016/j.pmedr.2022.102073 (PMC9722442; doi:10.1016/j.pmedr.2022.102073)
Supplement: Supplementary data 12 [file mmc12.docx]

**Table S1**. Interview codes, definitions and comments by school staff, parents and adolescents

| Code | Code Definition | # school staff who said this/# times | | # parents who said this/# times | | #adolescents who said this/# times | |
| --- | --- | --- | --- | --- | --- | --- | --- |
| Personal experience with mental illness - Yes | indicates that they have had personal experience (themselves, friend or family member) with mental illness | N/A | N/A | 3 | 3 | 4 | 4 |
| Personal experience with mental illness - No | indicates that they have not had personal experience (themselves, friend or family member) with mental illness | N/A | N/A | 1 | 1 | 3 | 3 |
| Personal experience with mental illness - Don't know | indicates that they maybe have had personal experience (themselves, friend or family member) with mental illness | N/A | N/A | 0 | 0 | 0 | 0 |
| Recall 19.20 Screener - Yes | indicates that they remember the screener | N/A | N/A | 4 | 4 | 6 | 7 |
| Recall 19.20 Screener - No | indicates that they do not remember the screener | N/A | N/A | 1 | 1 | 0 | 0 |
| Recall 19.20 Screener - Don’t know | indicates that they might remember the screener | N/A | N/A | 0 | 0 | 1 | 3 |
| Appropriateness of a Screener in school - Yes | indicates that they believe a depression screener is appropriate in school | N/A | N/A | 4 | 5 | N/A | N/A |
| Appropriateness of a Screener in school - No | indicates that they believe a depression screener is not appropriate in school | N/A | N/A | 0 | 0 | N/A | N/A |
| Appropriateness of a Screener in school - Don't know | indicates that theyare unsure whether or not a depression screener is appropriate in school | N/A | N/A | 0 | 0 | N/A | N/A |
| Steps if positive for depression | Steps school should take if child is screened positive for depression | N/A | N/A | 4 | 11 | 7 | 14 |
| Steps if positive for suicide risk | Steps school should take if child is screened positive for suicide | N/A | N/A | 4 | 10 | N/A | N/A |
| Parent steps for depression | Steps a parent will take if their child might be depressed | N/A | N/A | 4 | 8 | N/A | N/A |
| Parent steps for suicide | Steps a parent will take if their child might be suicidal | N/A | N/A | 2 | 3 | N/A | N/A |
| Location or context of conversation | discussion about the context (setting, time, place, etc.) for a conversation with child about concern of depression or mental health | N/A | N/A | 4 | 9 | 7 | 37 |
| Words used in conversation | specific words or phrases used or description of how the conversation would be approached with child | N/A | N/A | 3 | 5 | 6 | 14 |
| Action steps -Unsure | comments regarding not knowing what the next step would be | N/A | N/A | 1 | 2 | 2 | 2 |
| Action steps - Treatment | comments regarding treatment for depression, length of time it takes for treatment for mental health | N/A | N/A | 4 | 13 | 7 | 50 |
| Resource suggestions | mention of any resources that might be helpful for conversations about depression with a teen | N/A | N/A | 4 | 9 | N/A | N/A |
| Adult trust title | comments indicating who they would trust to see screening results | N/A | N/A | N/A | N/A | 6 | 15 |
| adult trust what adult should do | comments indicating steps the adult should take with the screening results | N/A | N/A | N/A | N/A | 4 | 5 |
| Topics discussed with trusted adult | description of what should be talked about (i.e. counseling, medication, resources, etc) | N/A | N/A | N/A | N/A | 6 | 29 |
| Follow-up process - Feelings about parent knowing | (cognitive) about their parents knowing their positive screening result | N/A | N/A | N/A | N/A | 7 | 24 |
| Follow-up process - Reaction | (behaviors) steps they might take | N/A | N/A | N/A | N/A | 5 | 12 |
| Thoughts about counseling inside school | comments about counseling services in school | N/A | N/A | N/A | N/A | 3 | 4 |
| Thoughts about counseling outside of school | comments about counseling services outside of school | N/A | N/A | N/A | N/A | 5 | 11 |
| Future plans to continue screening students for depression | explanation as to why and what the screening would look like | 5 | 15 | N/A | N/A | N/A | N/A |
| Future plans to not continue screening students for depression | explanations or reasons why not continuing screening | 5 | 13 | N/A | N/A | N/A | N/A |
| Adequate staffing for student follow-up challenges | any comments about challenges to have enough staff for follow-up with students on day of the screening (i.e. suicidal) or shortly after | 8 | 14 | N/A | N/A | N/A | N/A |
| Other challenges | any comments about challenges not including staffing | 7 | 19 | N/A | N/A | N/A | N/A |
| Facilitators | any comments describing components of the screener or follow-up that went smoothly (i.e. not a challenge). | 4 | 7 | N/A | N/A | N/A | N/A |
| School Staff and admin Screen | titles of individuals who should be involved in the screener | 4 | 6 | N/A | N/A | N/A | N/A |
| School Staff and admin Follow-up | titles of individuals who should be involved in the follow-up process | 7 | 8 | N/A | N/A | N/A | N/A |
| School staff and admin screen or follow-up | titles of individuals who should be involved in the screening and follow-up process but not specified as to which individuals should be involved in either | 5 | 8 | N/A | N/A | N/A | N/A |
| Methods of communication | description of how the school effectively worked together or communicated (i.e. committee, meetings, email, etc) | 10 | 18 | N/A | N/A | N/A | N/A |
| School access to screener responses | comments regarding student concern about school having access to responses | 11 | 29 | N/A | N/A | N/A | N/A |
| Two-week Suicide | thoughts on how the two-week time-period could be made clear | 11 | 20 | N/A | N/A | N/A | N/A |
| Management of Higher Numbers - New | explanations as to why and what a school’s NEW approach was to manage the higher number of students referred | 3 | 12 | N/A | N/A | N/A | N/A |
| Management of Higher Numbers - Same | explanations as to why and what a school’s SAME approach was to manage the higher number of students referred | 9 | 26 | N/A | N/A | N/A | N/A |
| Management of Higher Numbers - Unspecified | unclear whether the approach was new or the same | 0 | 0 | N/A | N/A | N/A | N/A |
| Peer-to-peer support group | Feedback on a school-based support group for mental health | 11 | 27 | N/A | N/A | N/A | N/A |
| In-school is more feasible for students | comments regarding students using in-school counseling because it’s easier/more convenient | 10 | 13 | N/A | N/A | N/A | N/A |
| In-school is an option for less serious needs | comments regarding students using in-school counseling when it’s a simpler case that doesn’t require a more advanced outside therapist. | 1 | 1 | N/A | N/A | N/A | N/A |
| Other reasons | comments regarding students using in-school counseling for other reasons not previously listed | 2 | 2 | N/A | N/A | N/A | N/A |
| Immediate score calculation and resources | feedback on the feasibility and effectiveness of implementing a screener that calculates scores after students complete it and offers resources | 11 | 17 | N/A | N/A | N/A | N/A |
| Screening after learning unit on mental health | feedback on the feasibility and effectiveness of implementing a screener after a unit of mental health in a health class or mental health club | 11 | 22 | N/A | N/A | N/A | N/A |
| Other ideas to incorporate screening | feedback on the feasibility and effectiveness of implementing a screener in ideas shared by the interviewee | 9 | 22 | N/A | N/A | N/A | N/A |
| Preferred grade to screen | feedback regarding which grade should be prioritized for a mental health screen | 11 | 26 | N/A | N/A | N/A | N/A |
